# miR-936 Suppresses Cell Proliferation, Invasion, and Drug Resistance of Laryngeal Squamous Cell Carcinoma and Targets GPR78

**DOI:** 10.3389/fonc.2020.00060

**Published:** 2020-02-04

**Authors:** Xi-Jun Lin, Hui Liu, Pei Li, Hai-Feng Wang, An-Kui Yang, Jin-Ming Di, Qi-Wei Jiang, Yang Yang, Jia-Rong Huang, Meng-Ling Yuan, Zi-Hao Xing, Meng-Ning Wei, Yao Li, Zhi Shi, Jin Ye

**Affiliations:** ^1^Department of Otolaryngology-Head and Neck Surgery, The Third Affiliated Hospital, Sun Yat-sen University, Guangzhou, China; ^2^Division of Pulmonary and Critical Care, Department of Internal Medicine, The Third Affiliated Hospital, Sun Yat-sen University, Guangzhou, China; ^3^Department of Head and Neck, Sun Yat-sen University Cancer Center, Guangzhou, China; ^4^State Key Laboratory of Oncology in South China, Guangzhou, China; ^5^Collaborative Innovation Center for Cancer Medicine, Guangzhou, China; ^6^Department of Urology, The Third Affiliated Hospital, Sun Yat-sen University, Guangzhou, China; ^7^Department of Cell Biology & Institute of Biomedicine, National Engineering Research Center of Genetic Medicine, Guangdong Provincial Key Laboratory of Bioengineering Medicine, College of Life Science and Technology, Jinan University, Guangzhou, China

**Keywords:** laryngeal squamous cell carcinoma, miR-936, GPR78, proliferation, invasion, drug resistance

## Abstract

MicroRNAs (miRs) play important roles in tumor progression. miR-936 has been reported to suppress cell invasion and proliferation of glioma and non-small cell lung cancer. Nevertheless, the function of miR-936 in laryngeal squamous cell carcinoma (LSCC) remains undiscovered. Hence, our study was to investigate the role of miR-936 in LSCC. In our present research, we have testified that miR-936 was substantially downregulated in LSCC tissues compared with adjacent normal tissues. Furthermore, miR-936 could inhibit proliferation, migration and invasion, and improve the sensitivity to doxorubicin and cisplatin of LSCC cells. Additionally, luciferase reporter assays were performed to confirm that GPR78 was a novel target of miR-936, and the protein expression of GPR78 was obviously inhibited by miR-936 in LSCC cells. In summary, our study indicates that the miR-936/GPR78 axis could be both a diagnostic marker and a therapeutic target for LSCC.

## Introduction

Laryngeal cancer ranks at the fourteenth most universal type of cancer in the world (1). It is estimated there over 13,000 new laryngeal cancer cases and 3,000 death cases took place in the United States, while 26,000 new cases and 14,000 deaths in China (1–3). Laryngeal squamous cell carcinoma (LSCC) occupies 85–90% of total malignant tumors in the larynx (4, 5). Despite advances in diagnosis and treatment, the long-term prognosis of LSCC patients has not been satisfactory in the past 20 years (6–8). Accordingly, a profound apprehension of the molecular biological mechanisms involving in LSCC tumorigenesis and development is imminently needed.

MicroRNAs (miRs) are a classical non-coding small RNA playing crucial regulatory roles in diverse pathological and physiological progresses by a posttranscriptional mechanism through binding the 3′-untranslated regions (3′-UTR) of target genes (9–12). And miRs can act as tumor oncogenes or suppressors through regulating their target genes that are usually dysregulated in cancer (13–15). In our current study, we have proven that miR-936 expression profile is meaningfully reduced in LSCC specimens, and overexpression of miR-936 suppresses LSCC cells proliferation migration and invasion. Moreover, our results have further exhibited that GPR78 is a direct target of miR-936.

## Materials and Methods

### Patients and Specimens

Twenty-five cases of LSCC and matching normal tissues were acquired from patients at the Department of Otolaryngology-Head and Neck Surgery, the Third Affiliated Hospital, Sun Yat-sen University and the Department of Head and Neck, Cancer Center, Sun Yat-sen University between December 2013 and February 2017. All patients were diagnosed as LSCC for the first time who underwent total or partial laryngectomy without chemical therapy or neoadjuvant radical before and after surgery. Signed informed approvals were acquired from patients, and the study was approved by the ethics committee of the Third Affiliated Hospital, Sun Yat-sen University.

### Cell Culture and Reagents

The human LSCC cell line Hep-2, the normal bronchial epithelium cell line 16HBE and the HEK293T were ordered from China Center for Type Culture Collection (CCTCC). The human LSCC cell line KB-3-1 was kindly provided by Dr. Zhesheng Chen (St. John's University, USA). The cells were cultured at 37°C with 5% CO_2_ in a humidified atmosphere in Dulbecco's modified Eagle's medium (DMEM) containing 100 Unit/ml penicillin, 100 ng/ml streptomycin and 10% fetal bovine serum (FBS). Cell lines applied in this project were authenticated by short tandem repeat fingerprinting <3 months when this study was started. Anti-GPR78 (AB61731a) was from Sangon Biotech. The antibody of anti-GAPDH (KM9002) was purchased from Tianjin Sungene Biotech.

### Plasmid

The synthesized precursor hsa-miR-936 was cloned into lentiviral vector pLKO.1-GFP to generate the hsa-miR-936 lentivirus construct. The GPR78 3′UTR fragment was cloned into a psiCHECK-2 dual luciferase reporter construct. Lentivirus was packaged with HEK293T cells and harvested from the supernatant of medium. Stable cell line was obtained through infecting lentivirus in Hep-2 or KB-3-1 cells and selecting with puromycin.

### RNA Extraction and Real-Time Quantitative PCR (RT-qPCR)

Total RNAs were extracted from cells and tissues by applying HiPure Total RNA Mini Kit (Magen). Reverse transcription was performed with HiFi-script cDNA kit (Cwbio) according to the instruction. The Bestar^TM^ Real time PCR Master Mix was applied for RT-qPCR by SYER Green Method. All reactions were carried out in triplicate and repeated at least three independent times. The results of RT-qPCR were normalized to U6 by applying the 2^−ΔΔCt^ method. The following primers were ordered from Sangon Biotech: miR-936 forward: 5′-AACGAGACGACGACAGAC-3′; miR-936 reverse: 5′-ACAGTAGAGGGAGGAATCGCAG-3′; U6 forward: 5′- GCGCGTCGTGAAGCGTTC-3′; U6 reverse: 5′- GTGCAGGGTCCGAGGT-3′ (16).

### Western Blot Analysis

Cells were washed with PBS, resuspended and lysed in RIPA buffer containing protease inhibitors (0.03% aprotinin, 10 ng/ml PMSF, 1 μM sodium orthovanadate, 1% NP-40, 0.1% SDS and 0.5% sodium deoxycholate,) at 4°C for 30 min. After centrifuging at 14,000 × g for 10 min, lysate supernatants were collected and stored at −80°C. Proteins were isolated by 12% SDS-PAGE gels and transferred to the membrane of polyvinylidene difluoride. After that, membrane was blocked by 5% BSA for 1 h, then incubated with the primary antibody and secondary antibody, successively. According to instruction, signal was measured through the chemiluminescent gel imaging system of ChemiDoc XRS (Bio-RAD) (17, 18).

### MTT Assay

Cells plated in 96-well plate were incubated for 0, 1, 2, 3 days, then add MTT 0.5 mg/ml. After 4 h incubation at 37°C, carefully absorb the culture medium in the well to prevent the cells from taking away formazan crystals, and dissolved crystals with 100 μl of DMSO. Multiscan Spectrum (Thermofisher) was used to measure the absorbance at 570 nm (19, 20).

### Wound Healing Assay

Cells were cultured in 6-well plate. Until the cells reached 80–90% confluence, using a sterile 10 μl pipette tip to draw a straight mark on the cell monolayer. After drawing, the delineated cells were washed and incubated in serum-free medium. The gap of wounds were measured by microscopic photograph at a certain time (21).

### Transwell Assay

Cells were plated in the upper compartment containing matrigel-coated polycarbonate membrane filter of a modified Boyden chamber (Corning), and the lower chamber was plated complete medium, and allowed to migrate for 24 h. Wiped the cells on the upper surface of membrane and fixed the cells on the lower surface of membrane by 4% paraformaldehyde and stained by 0.1% crystal violet staining solution (22).

### Luciferase Reporter Assay

The GPR78 mutated and wild-type (WT) 3′-UTR fragments were cloned into psiCHECK-2 reporter. HEK293T cells were plated in 24-well plates and co-transfected with mutated or WT 3′-UTR luciferase reporter and pLKO.1-GFP-miR-936. After 24 h, according to a protocol, cell lysates were obtain, and Firefly/Renilla luciferases ratios were measured with the Dual Luciferase Reporter Assay Kit (Promega) (23).

### Statistical Analysis

All statistics were analyzed by SPSS 20.0, and the results were shown as mean ± SD or median with the interquartile range. The Student's *t*-test and Mann–Whitney U-test were applied to analyze comparisons of two groups, and one-way ANOVA and Kruskal-Wallis test were applied to analyze comparisons of multiple groups. The *P* < 0.05 were considered statistically significant.

## Results

### Downregulation of miR-936 in LSCC Is Correlated With Differentiation, Lymph Node Metastasis and T Stages

**Figure 1:**
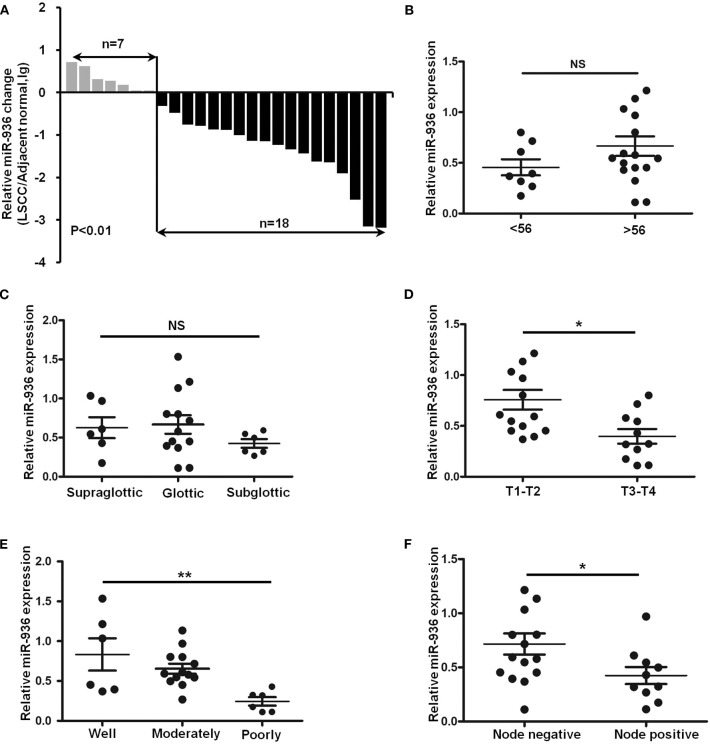
Downregulation of miR-936 in LSCC is correlated with T stages, differentiation and lymph node metastasis. **(A)** Expression of miR-936 in 25 pairs of LSCC tissues and adjacent normal tissues was detected using RT-qPCR. The relative miR-936 expression in two groups of LSCC tissues classified by age **(B)**, T stage **(D)**, and lymph node metastasis **(F)** were analyzed with Mann-Whitney *U*-test. The relative miR-936 expression in three groups of LSCC tissues classified by differentiation **(C)** and primary location **(E)** were analyzed with Kruskal-Wallis test. Data are presented as mean ± SD or median with the interquartile range. *p < 0.05; **p < 0.01; NS, no statistical significance.

To explore miR-936 expression in the LSCC tissues, RT-qPCR was used to check with 25 pairs of laryngeal cancer and normal tissue. Results suggested that miR-936 expression was meaningfully downregulated in LSCC, with 72% (18/25) of the tumor tissues showing reduced expression compared to matched normal controls (Figure 1A). Further, we found that miR-936 expression was correlated with tumor grade, lymph node metastasis and T Stages, but not correlated with tumor primary locations and age. The expression of miR-936 in negative lymph node metastasis, well-differentiation and T1-2 groups were higher than that in positive lymph node metastasis, poor differentiation, and T3-4 groups respectively (Table 1 and Figures 1B–F). According to these data, the progression of LSCC may be associated with miR-936 expression.

**Table 1:** Relationship between miR-936 expression level and clinicopathologic parameters.

**Characteristics (*n*)**	**miR-936 level^a^**	***P*-value^b^**
Age		0.0857
<56 (8)	0.4560 ± 0.2251	
≥56 (17)	0.6663 ± 0.3930	
T classification		0.0092
T1-2 (14)	0.7573 ± 0.3623	
T3-4 (11)	0.3976 ± 0.2368	
Primary location		0.3969
Supraglottic (6)	0.6266 ± 0.3269	
Glottic (13)	0.6668 ± 0.4270	
Subglottic (6)	0.4246 ± 0.1377	
Differentiation		0.0066
Well (6)	0.8329 ± 0.4957	
Moderately (6)	0.6546 ± 0.2286	
Poorly (13)	0.2446 ± 0.1313	
Lymph node metastasis		0.0315
Negative (14)	0.7273 ± 0.3892	
Positive (11)	0.4357± 0.2389	

a *Scores determined by qRT-PCR in mean ± SD*.

b *Student's T- test (for 2 groups) or one way ANOVA (for > 2 groups)*.

### Overexpression of miR-936 Suppresses the Proliferation of LSCC Cells

**Figure 2:**
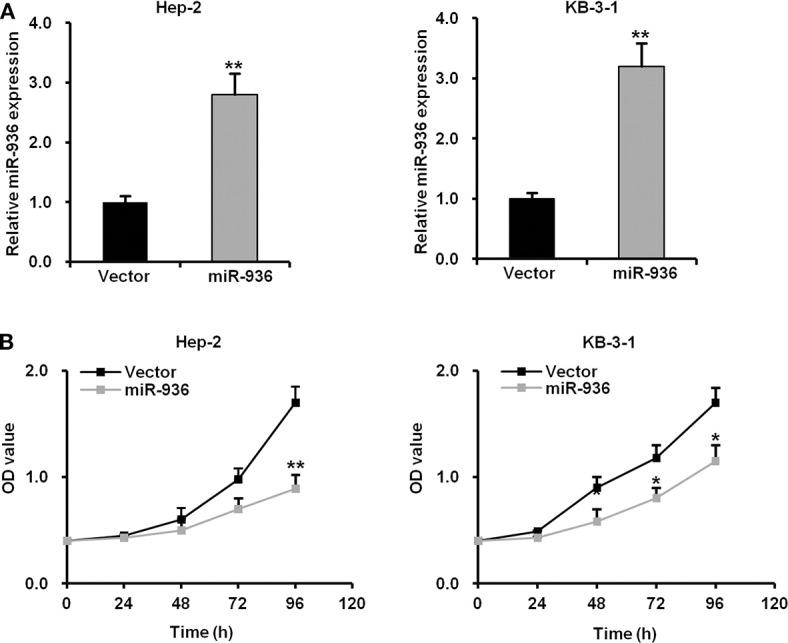
Overexpression of miR-936 suppresses the proliferation of LSCC cells. **(A)** RT-qPCR analysis of the relative miR-936 expression in Hep-2 and KB-3-1 cells expressed vector control and miR-936. **(B)** Cell proliferation of the two LSCC cell lines was measured using the MTT assay. OD values were measured every 24 h for 96 h with or without miR-936 transfection. Data are presented as mean ± SD. Student's *t*-test was used for statistical analysis. *p < 0.05; **p < 0.01.

To investigate the function of miR-936 in LSCC, Hep-2 and KB-3-1 cells were infected with lentivirus expressing precursor miR-936, which successfully upregulated miR-936 in the cells (Figure 2A). The growth curves determined by MTT assay indicates that the proliferation abilities of Hep-2 and KB-3-1 cells were significantly attenuated when miR-936 was overexpressed (Figure 2B).

### Overexpression of miR-936 Suppresses the Migration and Invasion of LSCC Cells

**Figure 3:**
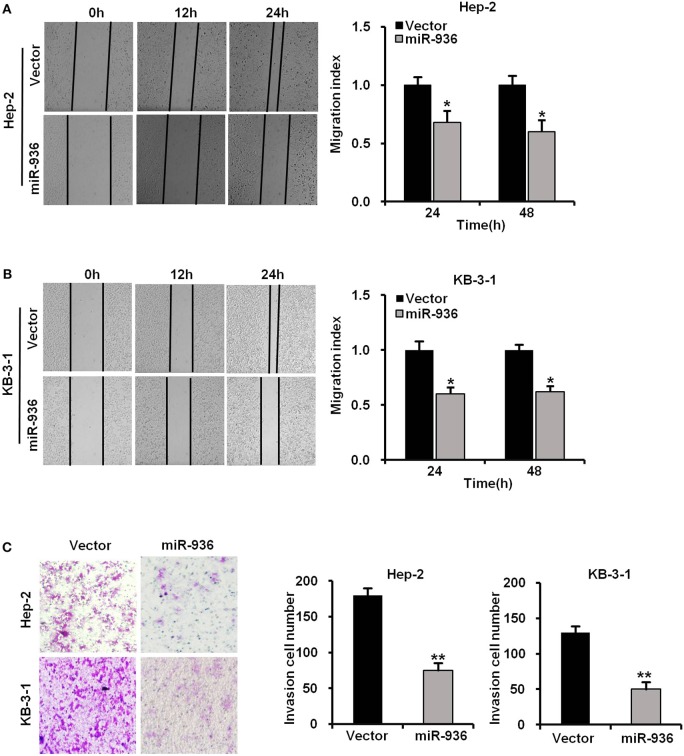
Overexpression of miR-936 suppresses the migration and invasion of LSCC cells. **(A,B)** Representative images and quantification of the indicated cells migration as determined with wound healing assay. **(C)** Representative images and quantification of the indicated cells invasion as determined with Transwell assay. Data are presented as mean ± SD. Student's *t*-test was used for statistical analysis. *p < 0.05; **p < 0.01.

To further verify whether miR-936 has an influence on the migration and invasion of LSCC cells, we performed wound healing and transwell assays in Hep-2 and KB-3-1 cells with miR-936 overexpression. The outcomes revealed that the migration and invasion of miR-936 overexpressing cells were importantly decreased when compared with control cells (Figures 3A–C).

### Overexpression of miR-936 Improves the Drug Sensitivity of LSCC Cells to Doxorubicin and Cisplatin

**Figure 4:**
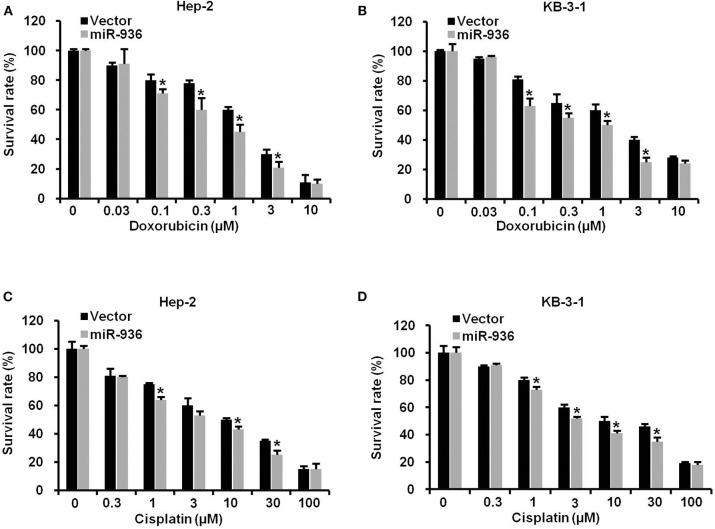
Overexpression of miR-936 improves the drug sensitivity of LSCC cells to doxorubicin and cisplatin. **(A–D)** Cell survival of the indicated cells treated with doxorubicin and cisplatin as determined with MTT assay. Data are presented as mean ± SD. Student's *t*-test was used for statistical analysis. *P < 0.05.

To verify the effect of miR-936 on LSCC cells treated with chemotherapy drugs, we treated indicated cells with doxorubicin or cisplatin in different concentrations. As shown in Figures 4A–D, the drug resistance to doxorubicin or cisplatin was significantly lower in cells overexpressing miR-936 in comparison with control groups in Hep-2 and KB-3-1 cells. These data suggested that increasing miR-936 expression could improve the drug sensitivity of LSCC cells to chemotherapeutic drugs.

### miR-936 Directly Targets GPR78

**Figure 5:**
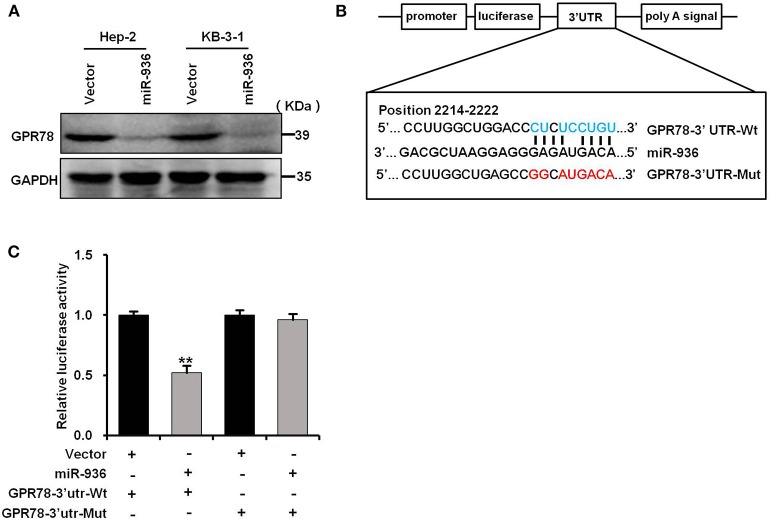
miR-936 directly targets GPR78. **(A)** Western blot analysis of GPR78 protein expressions in the indicated cells. GAPDH is the loading control. **(B)** A schematic diagram of the reporter constructs showed the wild type (Wt) and mutant (Mut) sequences of the miR-936 binding sites within human GPR78 3′-UTR. **(C)** Luciferase activity of reporters with GPR78 Wt or Mut 3′-UTR in the HEK293T cells. Data are presented as mean ± SD. Student's *t*-test was used for statistical analysis. **p < 0.01.

To understand the mechanism of miR-936 as a tumor suppressor in LSCC, we combined RNAhybird and PITA to search the new potential targets of miR-936. Both algorithms reveal that GPR78 was a downstream gene of miR-936. We then performed western blot analysis and found that overexpressing miR-936 in Hep-2 and KB-3-1 cells could decrease GPR78 protein levels notably (Figure 5A). The interaction between miR-936 and the 3′-UTR of GPR78 was illustrated in Figure 5B. And luciferase reporter assays were used in HEK293T cells to test whether miR-936 could directly interact with the 3′-UTR of GPR78. The ratio of fluorescence activity indicates the inhibitory effect of miR-936 on GPR78 in wild or mutant 3′-UTR. As exhibited in Figure 5C, overexpression of miR-936 markedly suppressed the luciferase activity of GPR78 WT 3′-UTR compared to mutant 3′-UTR. The result above indicated that miR-936 directly suppresses GPR78 expression through binding its 3′-UTR (2214nt~2222nt).

## Discussion

The development of tumors is a synergistic process of tumor-associated activation and inhibition genes. A growing number of studies have shown that abnormal expression of miRs happened in most types of human malignancies, including LSCC (24). In our present study, miR-936 was downregulated in LSCC tissues in comparison with matching normal tissues, and correlated with poor clinical features, suggesting that miR-936 might be associated with tumor progression in LSCC. Biological function experiments indicated that overexpression of miR-936 meaningfully inhibited the proliferation, migration, and invasion of LSCC cells. Furthermore, overexpression of miR-936 improved the drug sensitivity of LSCC cells to doxorubicin and cisplatin which currently are used for LSCC chemotherapy in clinic, indicating the significance of the findings that miR-936 sensitized the anticancer effects of doxorubicin and cisplatin in LSCC. Moreover, computerized algorithm predicted that GPR78 was a direct downstream target of miR-936, and that overexpression of miR-936 can effectively reduce GPR78 protein expression in LSCC cells. Previous researches have manifested that down-regulated miRs are often described as biomarkers or therapeutic targets in laryngeal squamous cell carcinoma. For instance, we recently reported t miR-194 works as a tumor suppressor in LSCC through targeting Wee1 (23). The expression of miR-375 is also reduced in LSCC tissues, and overexpression of miR-375 could reduce LSCC cell proliferation, motility and invasion by targeting IGF1R (25). In addition, miR-34a/c is also downregulated in LSCC tissues and inhibits LSCC cells proliferation by inducing cell cycle arrest through directly suppressing GALNT7 (26). While miR-27a is upregulated in LSCC specimens, and overexpression of miR-27a enhances LSCC cells proliferation by targeting PLK2 (27). MiR-93 is also upregulated in LSCC tissues and promotes the proliferation, migration, and invasion by inhibiting cyclin G2 (28).

Previous studies have been reported that miR-936 were down-regulated in glioma and induced cell cycle arrest via targeting CKS1 (29). Another report has also demonstrated that miR-936 could directly target E2F2 to inhibit the invasion and proliferation of non-small cell lung cancer cell (30). However, the biological functions of miR-936 have not been described in other tumors, including LSCC. Our study confirmed that miR-936 expression profile was downregulated in LSCC tissues, which indicates that miR-936 may be related to the progression of LSCC. Searching miRNA target genes is fundamental to understanding its carcinogenic regulatory mechanism and the effective molecular therapeutic targets. By bioinformatics algorithm and experiment validation, we testified that GPR78 was a direct target of miR-936. GPR78, an orphan G-protein coupled receptor, is situated in an area of chromosome 4p where have been shown connection to schizophrenia and bipolar affective dysfunction (31). Recently, GPR78 was testified highly expressed in lung cancer cell, and knockdown of GPR78 prominently suppressed cell migration and metastasis (32). However, the expression and function of GPR78 in LSCC need to be further investigated in the future.

In conclusion, our results proved that miR-936 attenuated the proliferation, migration and invasion of LSCC cells and targeted GPR78. Moreover, high level of miR-936 could improve the sensitivity of LSCC cells to doxorubicin and cisplatin. The results manifests that the miR-936/GPR78 axis could play as a novel biomarker and a therapeutic target of LSCC.

## Data Availability Statement

The datasets generated for this study are available on request to the corresponding author.

## Ethics Statement

The studies involving human participants were reviewed and approved by the ethics committee of The Third Affiliated Hospital, Sun Yat-sen University. The patients/participants provided their written informed consent to participate in this study.

## Author Contributions

X-JL, HL, PL, H-FW, ZS, and JY designed the projects, conducted the experiments, analyzed the results, and wrote the manuscript. A-KY, J-MD, Q-WJ, YY, J-RH, M-LY, Z-HX, M-NW, and YL conducted the experiments. All authors read and agreed the final manuscript.

### Conflict of Interest

The authors declare that the research was conducted in the absence of any commercial or financial relationships that could be construed as a potential conflict of interest.
